# Colonel Leslie Ogilby Patterson

**Published:** 1910-07-23

**Authors:** 


### Obituary.

The late Colonel Leslie Ogilby Patterson, of the Army Medical Service, whose death is announced, qualified as a member of the Royal College of Surgeons of England in 1851 and joined the Army in 1854. He served in the Burmese War in 1856, and was present at several actions during the Indian Mutiny, Jugdispur, and was wounded at Sirchooa. He was mentioned in despatches, awarded the Mutiny medal, and retired in 1880 with the honorary rank of Brigade-Surgeon. He had been at one time a surgeon to the Royal Canadian Rifle Regiment and a surgeon-major to the Royal Artillery.

